# A novel ferroptosis related gene signature is associated with prognosis in patients with ovarian serous cystadenocarcinoma

**DOI:** 10.1038/s41598-021-90126-5

**Published:** 2021-06-01

**Authors:** Zhixiang Yu, Haiyan He, Yanan Chen, Qiuhe Ji, Min Sun

**Affiliations:** 1 Department of Obstetrics and Gynecology, Tangdu Hospital, The Air Force Military Medical University, Xi’an, Shaanxi China; 2 Department of Endocrinology and Metabolism, Xijing Hospital, The Air Force Military Medical University, Xi’an, Shaanxi China; 3 Basic Medicine College, The Air Force Military Medical University, Xi’an, Shaanxi China; 4 Department of Medical Oncology, Jinling Hospital, School of Medicine,Nanjing University, Nanjing, Jiangsu, China

**Keywords:** Cancer, Biomarkers, Diseases, Oncology, Risk factors

## Abstract

Ovarian cancer (OV) is a common type of carcinoma in females. Many studies have reported that ferroptosis is associated with the prognosis of OV patients. However, the mechanism by which this occurs is not well understood. We utilized Genotype-Tissue Expression (GTEx) and The Cancer Genome Atlas (TCGA) to identify ferroptosis-related genes in OV. In the present study, we applied Cox regression analysis to select hub genes and used the least absolute shrinkage and selection operator to construct a prognosis prediction model with mRNA expression profiles and clinical data from TCGA. A series of analyses for this signature was performed in TCGA. We then verified the identified signature using International Cancer Genome Consortium (ICGC) data. After a series of analyses, we identified six hub genes (DNAJB6, RB1, VIMP/ SELENOS, STEAP3, BACH1, and ALOX12) that were then used to construct a model using a training data set. The model was then tested using a validation data set and was found to have high sensitivity and specificity. The identified ferroptosis-related hub genes might play a critical role in the mechanism of OV development. The gene signature we identified may be useful for future clinical applications.

## Introduction

Ovarian cancer is a severe threat to the health of females and is one of the gynecological cancers that can be fatal^1^. In some developed countries, such as the United States, it has been the top 5 causes of cancer-related death for females^2^. There are four main kinds of ovarian cancers, accounting for almost all advanced stage cases: ovarian serous cystadenocarcinoma (OV), peritoneal carcinoma, carcinosarcoma, and mixed carcinoma^3^. OV is the most common in clinical work and has been well-documented interpatiently^4^, intertumorally^5^, and intratumorally^6^. However, the mechanism is not well understood. The early symptoms of OV are latent, and the early diagnostic methods of OV are not mature, which leads to the fact that most OV is diagnosed at an advanced stage^7^. Due to the high heterogeneity of OV, the prognostic prediction seems challenging. Furthermore, there are huge differences between early-stage OV and advanced-stage OV in the treatment efficacy and prognosis^6^. Therefore, it is urgent to develop prognostic models.

Ferroptosis is a unique form of regulated cell death associated with iron metabolism^8^. The lethal accumulation of lipid peroxidation accelerates ferroptosis. Furthermore, Liang et al.^9^ reported that ferroptosis is involved in cancer apoptosis, which provides a potential therapeutic target for the treatment of malignancies. Ferroptosis-related genes can be divided into the following three groups: drivers, suppressors, and markers. Previous studies have reported that ferroptosis plays a crucial role in OV tumor-initiating cells in vivo^10^. Recent studies have revealed that some ferroptosis-related genes, including TFR1, IL6, and SCD1^11^, are correlated with OV development and apoptosis. However, there are still no studies about the relationship between ferroptosis-related genes and OV patient prognosis.

In this study, we used the mRNA expression data and clinical OV patient data from TCGA and IGCG. Moreover, we identified ferroptosis-related differentially expressed genes (DEGs) by comparing OV mRNA expression and regular ovarian tissue expression from GTEx. Then, we constructed a prognostic DEG signature with TCGA data and verified the multigene signature in an IGCG Australian OV patient cohort. After constructing the gene signature, we tested the model with Cox analysis to predict OV patient prognosis. Finally, we performed GO (Gene Ontology) enrichment analysis of the OV patient high-risk subgroup to explore the potential ferroptosis-related gene-associated pathways in OV.

## Methods

### Data collection

The mRNA expression data and corresponding clinical information of 379 OV patients were downloaded from TCGA (https://portal.gdc.cancer.gov/repository) on September 10, 2020. The mRNA expression data and corresponding clinical data of 88 normal ovarian tissues were downloaded from GTEx (https://gtexportal.org/) on September 10, 2020. We applied the normalization strategies offered in the "limma" R packages (https://bioconductor.org/packages/limma/)^12^ to the gene expression of the two different databases. The RNA-seq profiles and clinical data were acquired from the IGCG Australian OV patient cohort (https://dcc.icgc.org/releases/current/Projects/OV-AU) on September 15, 2020. We strictly obeyed the guidelines of the three databases.

We screened the FerrDb database (http://www.zhounan.org/ferrdb/) and obtained 259 ferroptosis-related genes^13^. These genes were grouped and are shown in Table 1.

**Table 1:** Group of the ferroptosis-related genes.

Data set	Gene count
Driver	108
Suppressor	69
Marker	111

There 28 genes repeat count more than one group.

### Construction and validation of a prognostic ferroptosis-related gene signature

After data normalization, we used the "limma" R package^12^ to identify the ferroptosis-related DEGs between OV in the TCGA cohort and normal ovarian tissues in GTEx. We set a false discovery rate (FDR) < 0.05 as a criterion. For primary screening, we performed a univariate Cox analysis of overall survival for each ferroptosis-related DEG. A *p* value < 0.05 was considered the cutoff for the genes with prognostic values. To avoid overfitting in the gene signature, we needed to simplify the signature as much as possible. The least absolute shrinkage and selection operator (LASSO) regression could offer a suitable choice for the selection of potential genes. We carried out LASSO regression with the DEGs, which was subjected to univariate Cox analysis using the R package "glmnet". The parameter λ decided the complexity of the model. Λ was defined as the penalty regularization, which was obtained at minimum partial likelihood deviance. Each patient's risk score was based on the LASSO analysis results and the expression of each gene. The risk score was calculated using the following formula: riskScore = ${e}^{sum(univariate Cox analysis*corresponding coefficient)}$. According to the median value, the patients were divided into a high-risk group and a low-risk group according to the signature they scored. We performed principal component analysis (PCA) and t-distributed stochastic neighbor embedding (tSNE) with the "stats" R package for the two groups. Moreover, we drew a series of pictures to visualize the differences between the two groups. Moreover, we constructed Kaplan–Meier (KM) survival plots to evaluate the prognostic value and receiver operating characteristic (ROC) curves with different time cutoffs to evaluate the predictive efficiency.

### Examination of the gene signature in another database

We applied the formula to the patients in the IGCG Australian OV patient cohort who had a clear outcome indicator five years after diagnosis. The patients were divided into high-risk and low-risk groups based on the median risk score. PCA and tSNE were also carried out in the next step. We performed similar tests to examine whether the model could determine the significance of the prognostic prediction.

### Functional enrichment and immune analysis

We performed Kyoto Encyclopedia of Genes and Genomes (KEGG)^14–16^ pathway enrichment analysis of data from TCGA and IGCG. Through pathway enrichment analysis, we explored the potential ferroptosis-related gene-associated pathways in OV development. Moreover, we calculated the infiltration score of 16 kinds of immune cells. We performed single-sample gene set enrichment analysis (ssGSEA) for further study of the 13 immune-related pathways. ssGSEA was finished with the help of the 'gsva' R package^17^.

### Verification of the clinical values of the signature

First, we carried out univariate Cox analysis for the risk score and other critical clinical factors to decide whether the risk score could be an independent prognostic factor. We took a risk score as a novel classification indicator for further research and compared several characteristics between the high-risk and low-risk groups. The above work was completed with the help of R software. A *p* value < 0.05 indicated that the difference was statistically significant.

## Results

A total of 379 OV patients were from the TCGA-OV cohort, and 82 OV patients were from the IGCG (OV-AUS) cohort. We downloaded the mRNA expression data and clinical files from both databases. Some samples were excluded for kinds of reasons that were not suitable for a further research.

### Identification of prognostic ferroptosis-related DEGs in TCGA cohort

**Figure 1:**
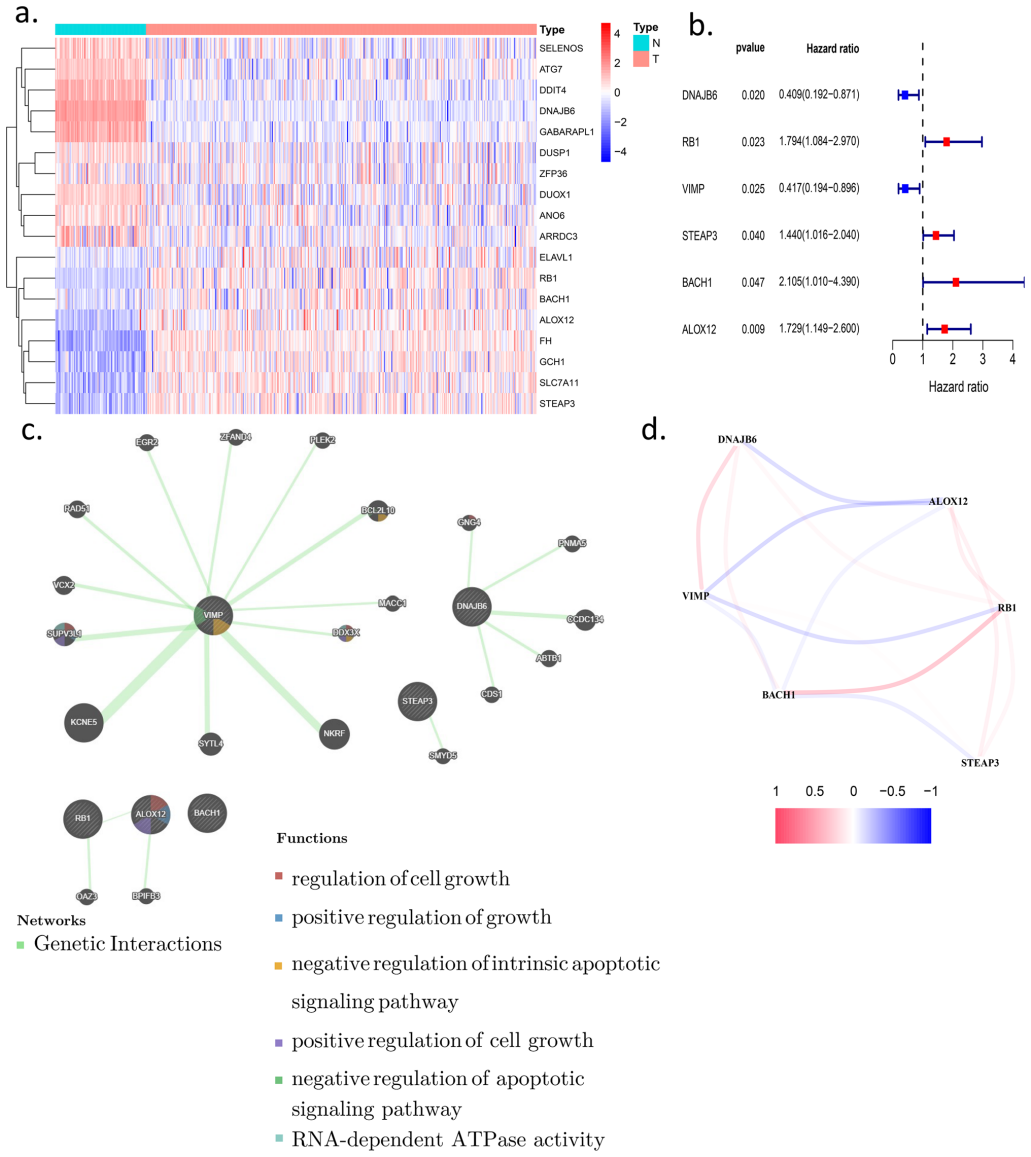
screening for the hub genes. (**a**) The heatmap of differently expressed genes in the normal and tumor group (**b**) the univariate Cox analysis of the genes equipped with a consistent tendency in mRNA expression in tumor samples and prognosis (**c**) the network of hubgenes and related genes with the function enrichment (**d**) the co-expression network between hub-genes..

DEGs from the comparison between OV and healthy ovaries accounted for most of the ferroptosis-related genes (199/259). We performed univariate Cox regression analysis for the DEGs to select prognostic ferroptosis-related DEGs. Eighteen genes passed the univariate Cox regression analysis with the cutoff of *p* < 0.05 (Fig. 1a). Six genes (DNAJB6, RB1, VIMP/SELENOS, STEAP3, BACH1, and ALOX12) had a consistent mRNA expression level in tumor samples and prognosis in the univariate Cox analysis (Fig. 1b). The other 12 genes were excluded from further study. First, we presented the correlation between the six genes (Fig. 1d). Then, we constructed the six preserved genes and associated gene network with the help of GeneMANIA (http://genemania.org/)^18^ in Fig. 1c. We performed the KM plot in the KM plotter (https://kmplot.com/analysis/index.php?p=service&cancer=ovar)^19^. DNAJB6, RB1, STEAP3, BACH1, and ALOX12 were associated with prognosis (supplementary figure S1), while VIMP/SELENOS might be a prognosis indicator in some cases (supplementary figure S2).

### Construction of a six-gene signature in TCGA cohort

**Figure 2:**
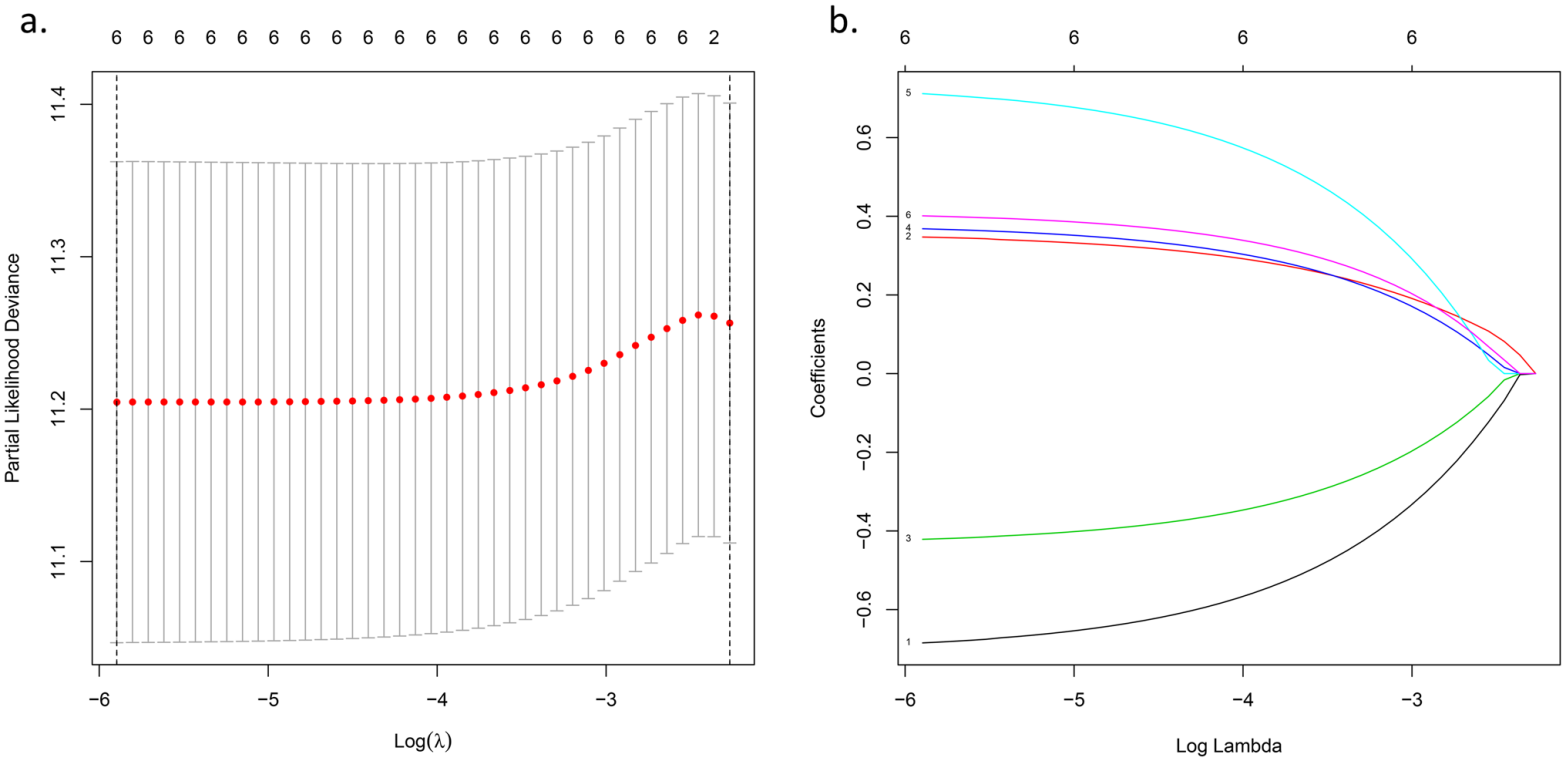
LASSO Cox regression analysis. (**a**) Partial likelihood deviance was plotted versus log (lambda). The vertical dotted line indicates the lambda value with the minimum error and the largest lambda value in which deviance was within one standard error of the minimum. (**b**) LASSO coefficient profiles of genes associated with survival of in patients with Ovarian cancer. LASSO, least absolute shrinkage and selection operator.

**Figure 3:**
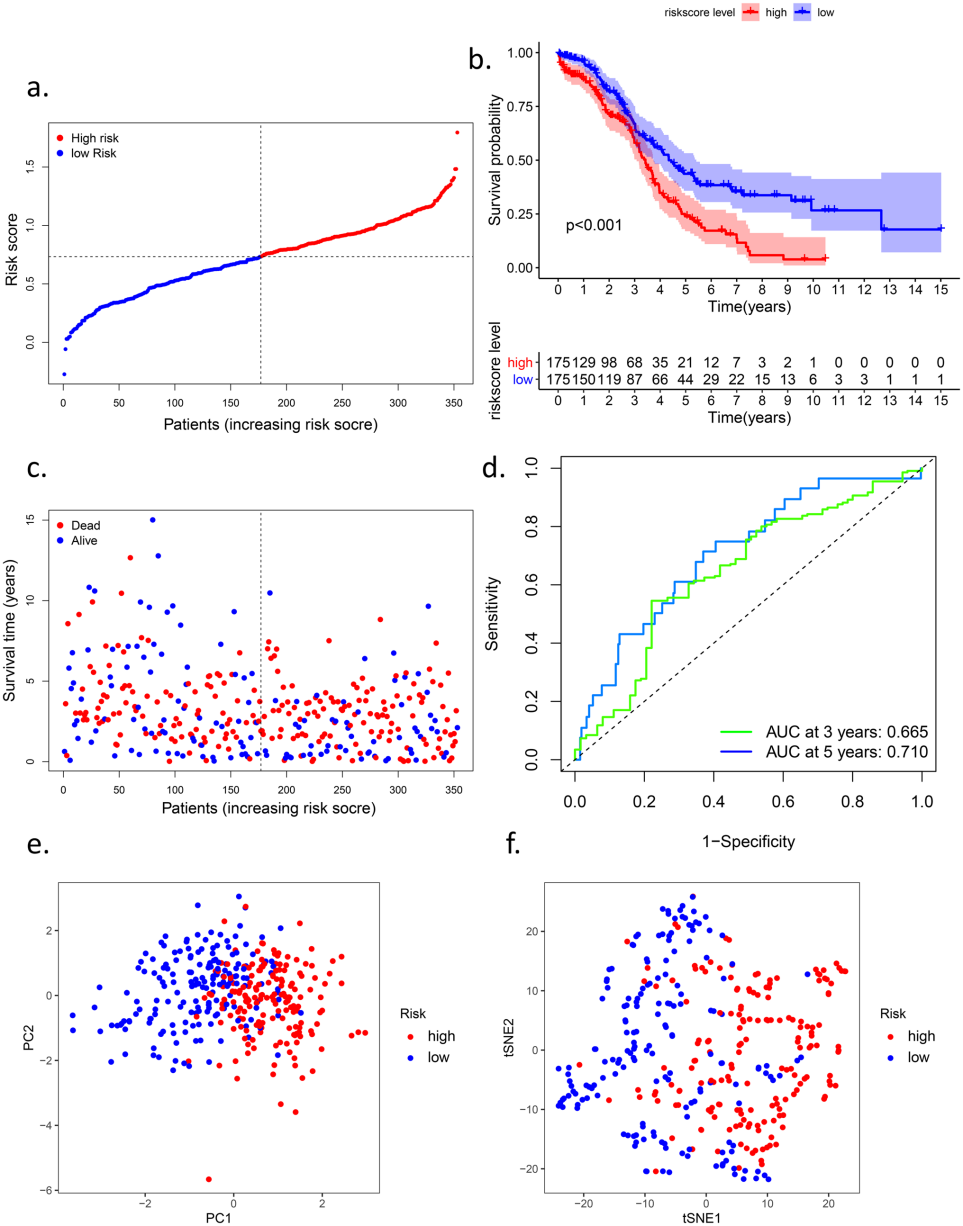
Prognostic analysis of the six-gene signature model in the TCGA cohort. (**a**) the distribution and median value of the risk scores in the TCGA cohort. (b) Kaplan-Meier curves for the OS of patients in the high-risk group and low-risk group in the TCGA cohort.. (**c**) the distributions of OS status, OS and risk score in the TCGA cohort. (**d**) AUC of time-dependent ROC curves verified the prognostic performance of the risk score in the TCGA cohort (**e**) PCA plot of the TCGA cohort. (**f**) t-SNE analysis of the TCGA cohort.

The more genes the signature included, the more complex the signature was. We performed LASSO Cox regression analysis of the six genes. All the genes were preserved after LASSO (Fig. 2b), and we constructed a six-gene signature based on the ideal λ (Fig. 2a). The risk score =$e$ (0.347*expression level of RB1 + 0.368*expression level of STEAP3 + 0.712*expression level of BACH1 + 0.401*expression level of ALOX12-0.685*expression level of DNAJB6-0.421*expression level of VIMP/ SELENOS). According to the median value of the risk score, we divided the patients into high-risk and low-risk groups (Fig. 3a). For further work, we drew a Kaplan–Meier curve (Fig. 3b) and a survival plot (Fig. 3c). Moreover, We listed some baseline information of patients in high- and low-risk groups (Table 2). We found that OV patients with high-risk scores had a higher death rate and a significantly worse overall survival (OS) than those with low-risk scores (supplementary figure S3). As for progression-free survival (PFS), representing the possible benefits for patients, patients in the high-risk group still suffered more from disease progression and death events (supplementary figure S5). The ROC curves showed the predictive value of the signature. The area under the curve (AUC) reached 0.602 at three years and 0.710 at five years (Fig. 3d), which indicated that the predictive performance of the signature worked well. The PCA and t-SNE analyses indicated that the signature could divide the OV patients into two groups (Fig. 3e,f). Further analysis of TCGA data is shown in the supplementary materials, the high-risk group was also associated with advanced TNM stage.

**Table 2:** Baseline information of the patients in high- and low-risk groups.

		High-risk	Low-risk	*p* value
Age	Media	59 (30–87)	58 (40–87)	0.6961
Grade	G1	1	0	0.294
	G2	21	16	
	G3	145	156	
	G4	1	0	
Stage	Stage I	0	1	0.8215
	Stage II	14	4	
	Stage III	127	146	
	Stage IV	32	23	
Tumor residual	< 10 mm	110	113	0.8092
	10–20 mm	16	8	
	> 20 mm	30	36	
Venous invasion	Yes	32	27	0.2727
	No	16	16	
OS	Events	107	87	**0.0312**
	Without events	67	88	
PFS	Events	109	91	**0.0444**
	Without events	65	84	

Bold values represents p<0.05 which means there exists significant differences bewteen the two groups

### Validation of the six-gene signature in the IGCG cohort

**Figure 4:**
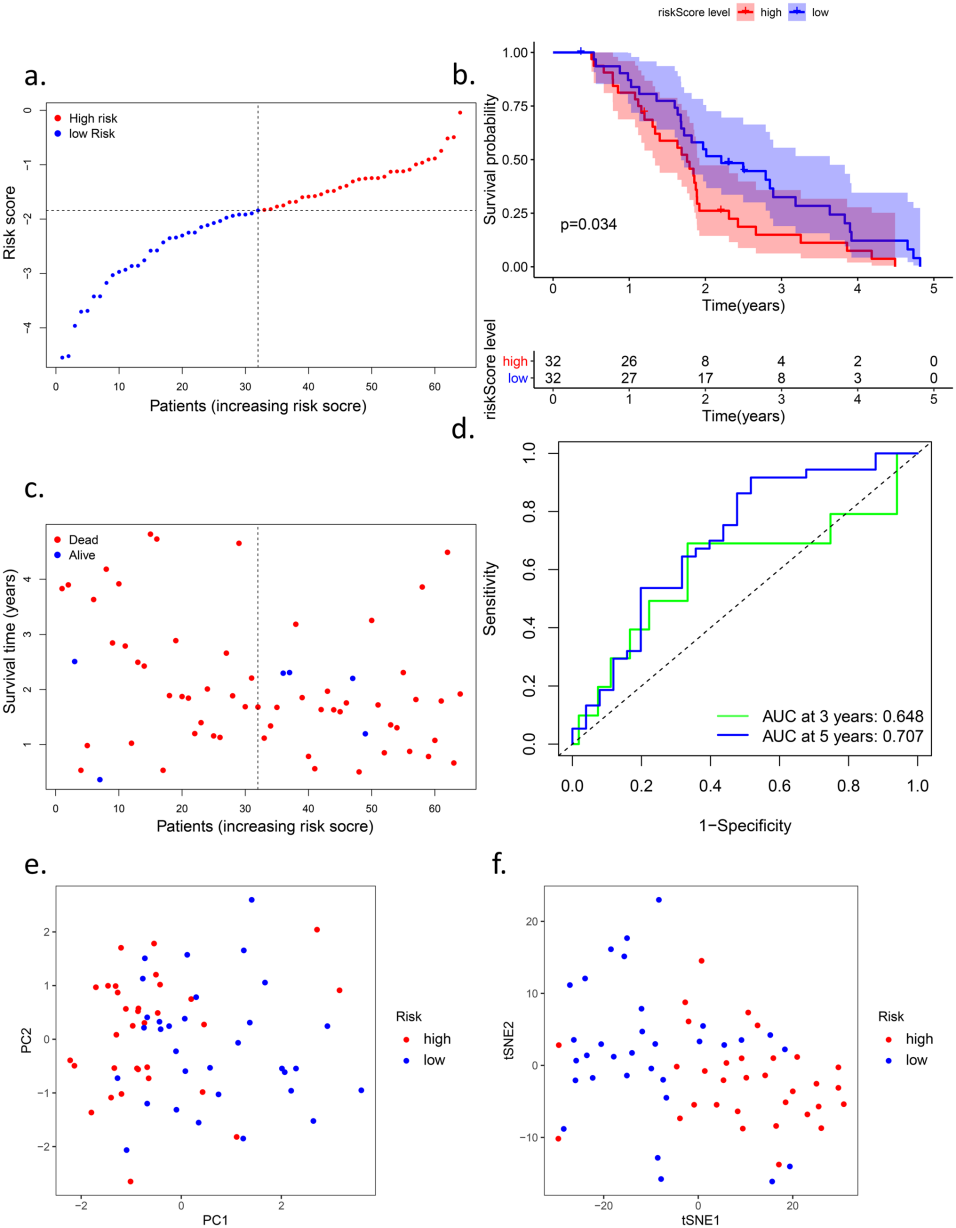
Validation of the six-gene signature in the ICGC cohort. (**a**) the distribution and median value of the risk scores in the ICGC cohort. (**b**) Kaplan-Meier curves for the OS of patients in the high-risk group and low-risk group in the ICGC cohort. (**c**) the distributions of OS status, OS and risk score in the ICGC cohort. (**d**) AUC of time-dependent ROC curves verified the prognostic performance of the risk score in the ICGC cohort. (**e**) PCA plot of the ICGC cohort. (**f**) t-SNE analysis of the ICGC cohort.

To ensure the robustness of the signature constructed in TCGA cohort, we applied the same formula mentioned above (Fig. 4). The patients who had a clear outcome in the five-year follow-up period were divided into high-risk and low-risk groups based on the median riskScore in the IGCG cohort (Fig. 4a). The PCA and t-SNE analyses were similar to the result of the TCGA cohort in that the signature could separate the two groups in opposite directions (Fig. 4e,f). Similarly, patients in the low-risk subgroup shared a better prognosis (Fig. 4b,c). The AUC of the six-gene signature in the IGCG was 0.648 at three years and 0.707 at five years (Fig. 4d). The test results mentioned above in the IGCG cohort revealed that the signature passed the examination.

### Independent prognostic and application values of the six-gene signature

**Figure 5:**
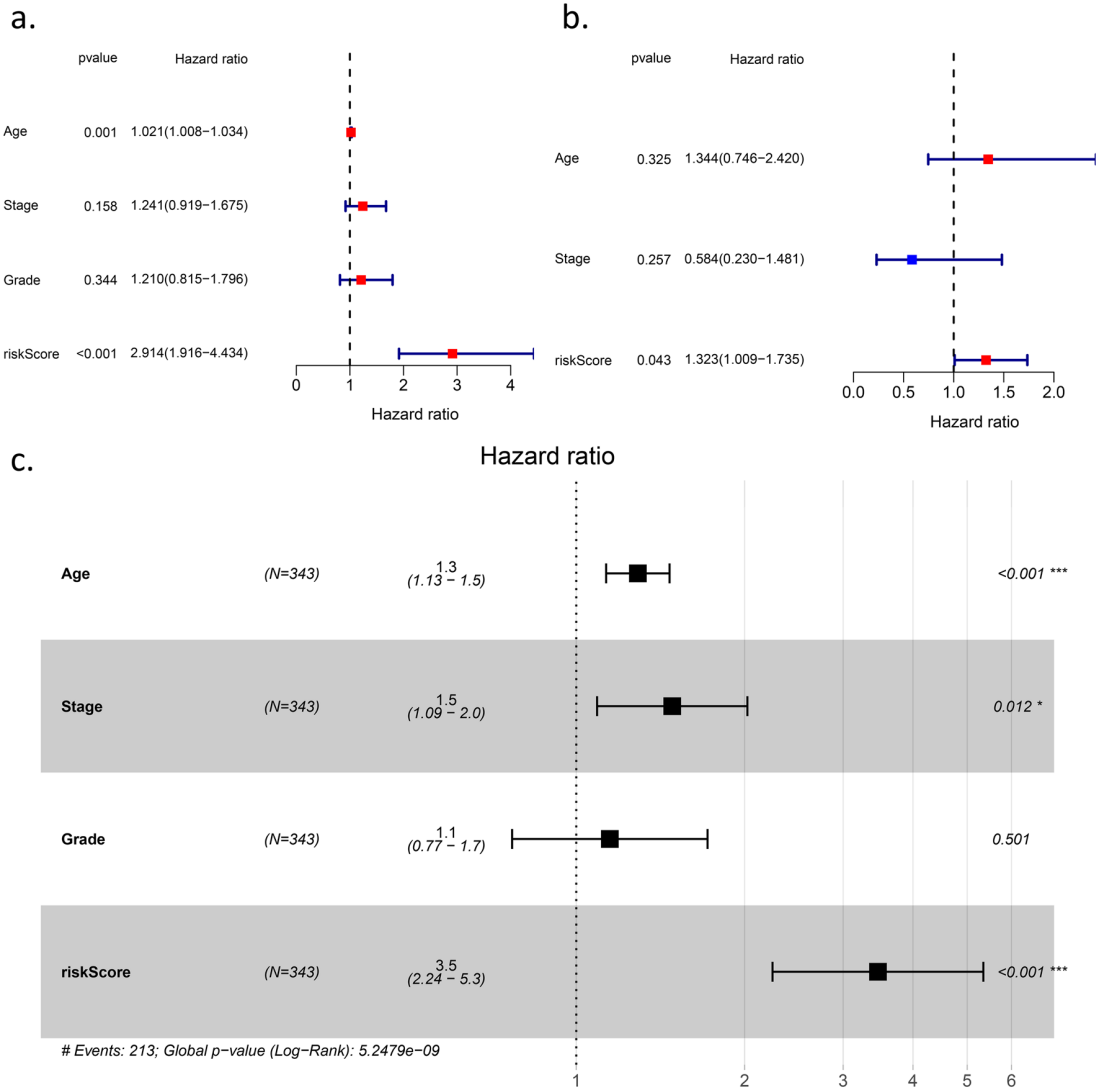
Results of the univariate Cox regression analyses regarding OS in the (**a**) TCGA derivation cohort and (**b**) the ICGC validation. (**c**) multivariate Cox regression analyses regarding OS in the TCGA derivation cohort.

**Figure 6:**
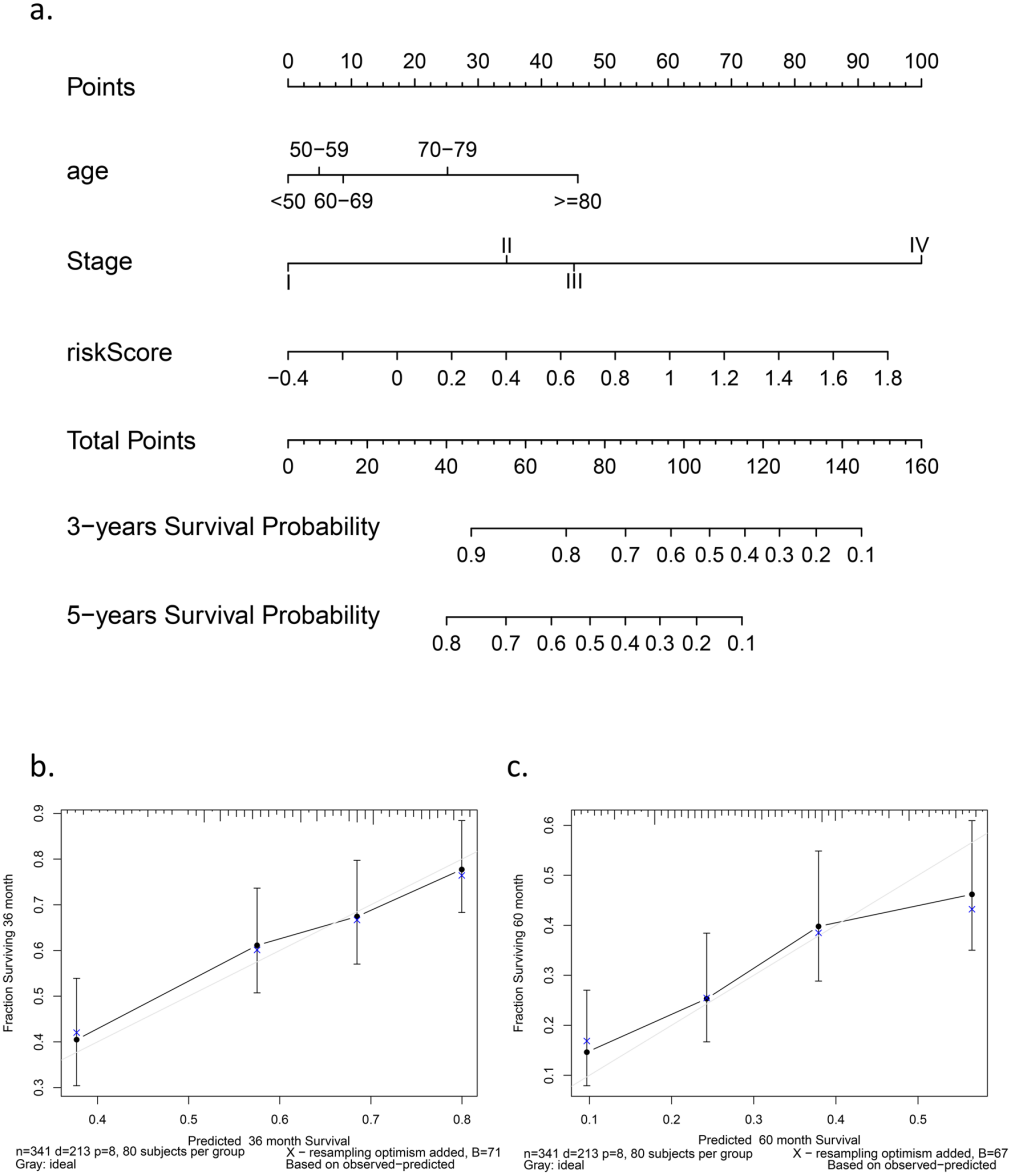
The application of the gene signature. (**a**) Nomogram to predict the survival of patients with OV(**b**) Calibration curve of the nomogram in 3 years. (**c**) Calibration curve of the nomogram in 5 years.

We carried out Cox regression analysis among the key variables in the clinical work and our signature to explore the application value (Fig. 5). In the univariate Cox regression analysis, the risk score was the only independent prognostic factor in TCGA (HR = 2.914 95% CI: 1.916 to 4.434, adj. *p* < 0.001) (Fig. 5a) and IGCG (HR = 1.323 95% CI: 1.009 to 1.735, adj. *p* = 0.043) (Fig. 5b). For further research, we performed multivariate Cox regression analysis for a series of available variables, including age, grade, and risk score (Fig. 5c). Moreover, we established a nomogram based on the multivariate Cox regression analysis results to predict the probability of OV patient survival at 3 and 5 years (Fig. 6a). We generated calibration curves to evaluate the nomogram and obtained an ideal match (Fig. 6b,c), which indicated that the model could be applied in the clinic.

### Gene enrichment analysis in the OV cohort

**Figure 7:**
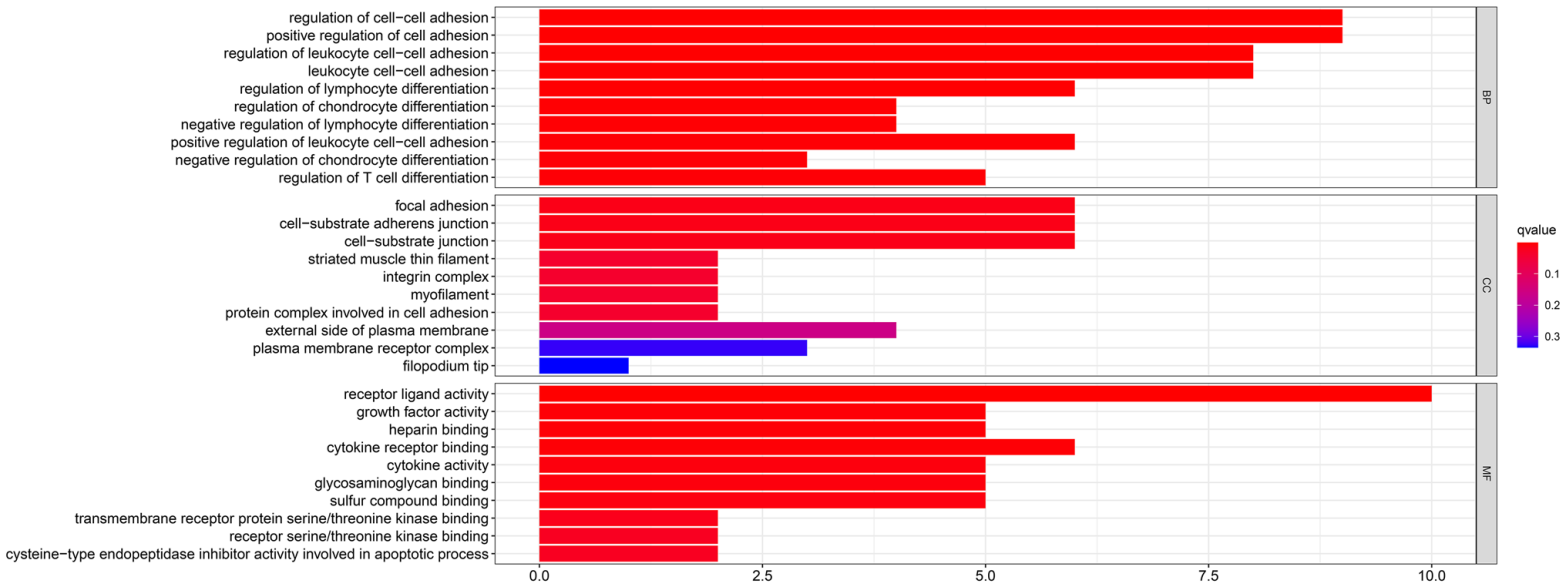
The GO (Gene Ontology) analysis for the differently expressed genes between high-risk and low-risk subgroups..

To determine the pathways and functions correlated with the risk score, GO (Gene Ontology) and KEGG analysis was carried out (Table 3). The GO analysis results indicated that the enrichment was associated with immunity, including "leukocyte cell–cell adhesion", "lymphocyte differentiation" and "T cell differentiation" (Fig. 7). The KEGG results were in Table 2^20–26^. And we had already got permission to use the KEGG software from the Kanehisa laboratory.

**Table.3:** KEGG enrichment analysis of the hub genes.

ID	Description	Gene ratio	*p* value	*p* adjust
hsa04060	Cytokine-cytokine receptor interaction^19^	6/27	3.74E−05	0.003406
hsa04810	Regulation of actin cytoskeleton^20^	5/27	0.000626	0.027426
hsa04151	PI3K-Akt signaling pathway^21^	6/27	0.00092	0.027426
hsa04550	Signaling pathways regulating pluripotency of stem cells^22^	4/27	0.001206	0.027426
hsa05140	Leishmaniasis^23^	3/27	0.002068	0.037644
hsa05410	Hypertrophic cardiomyopathy^24^	3/27	0.003227	0.048945
hsa05414	Dilated cardiomyopathy^25^	3/27	0.003873	0.050348

**Figure 8:**
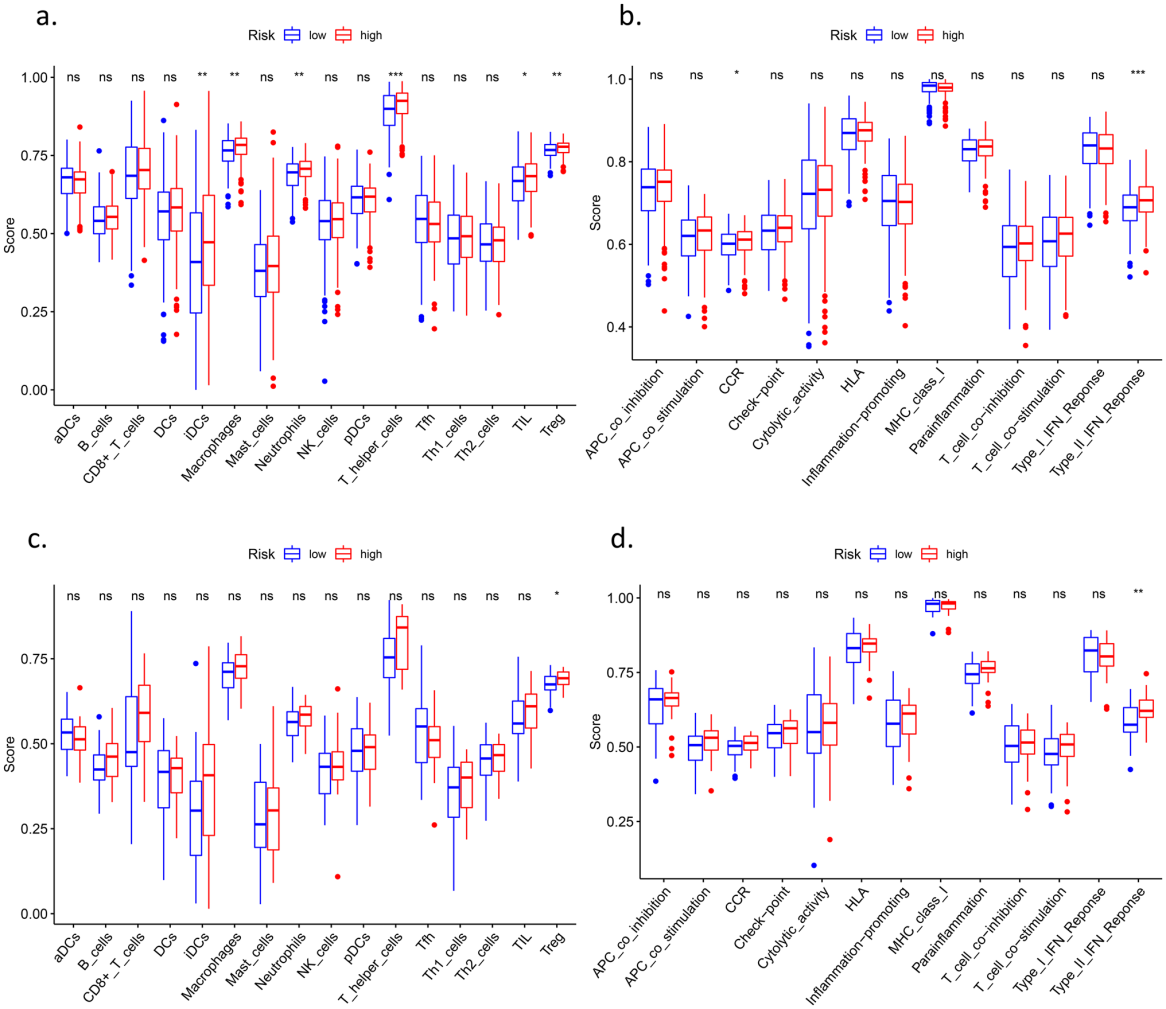
Comparison of the ssGSEA scores between different risk groups in the TCGA cohort (a, b) and ICGC cohort (c, d). The scores of 16 immune cells (a, c) and 13 immune-related functions (b, d) are displayed in boxplots. CCR, cytokine-cytokine receptor. Adjusted P values were showed as: ns, not significant; *, p < 0.05; **, p < 0.01; ***, p < 0.001.

To further explore the correlation between the signature and immune status, we quantified the enrichment scores of common immune cell subpopulations, related functions, or pathways with ssGSEA (Fig. 8). A series of immune cells were significantly different between the low-risk subgroup and high-risk subgroup in the TCGA cohort (adj. *p* < 0.05), which led to differences in immune functions, such as the type II IFN response and CCR (chemokine receptor). Treg and type II IFN responses were validated in the IGCG cohort (adj. *p* < 0.05), consistent with the GO analysis above.

## Discussion

Our study systematically researched ferroptosis-related genes in OV tumor tissues and their influence on prognosis. A novel six-gene signature was based on LASSO regression analysis, and the signature was validated with an external test set. We made full use of the value of the signature in predicting OS by nomogram and the KM-plots showed that our signature was a reliable factor for OV patients' prognosis both in OS and disease progression. Functional analyses indicated that the ferroptosis-related genes in OV were enriched in immune-related pathways.

Although a few previous studies have reported that several genes are associated with OV and some could be potential treatment targets, they did not pay enough attention to the correlation between ferroptosis-related genes and prognosis. To our surprise, most ferroptosis-related genes were significantly expressed between OV and healthy ovaries, which revealed that ferroptosis might play a key role in OV and the possibility of constructing a predictive signature with ferroptosis-related genes.

Six genes (DNAJB6, RB1, VIMP/ SELENOS, STEAP3, BACH1, and ALOX12) were involved in the model; these genes could be roughly divided into three groups: drivers (DNAJB6, BACH1 and ALOX12), suppressors (RB1) and markers (VIMP/ SELENOS and STEAP3).

BTB and CNC homology 1 (BACH1) is a ubiquitously expressed transcription factor. BACH1 plays a vital role in a series of pathways and biological processes, including oxidative stress, heme oxidation, the cell cycle, hematopoiesis, and immunity. Han et al.^27^ reported that high expression of BACH1 activates *p*-AKT and promotes ovarian cancer growth as a transcriptional regulator both in vitro and in vivo. Peng et al.^28^ and Rebbeck et al. ^29^ stated that BACH1 was involved in the BRCA1 damage response related to an increased risk of OV.

RB1 was reported to be related to defective DNA repair^30^. RB1 loss could lead to longer-term survival for OV patients. This verified the result that high expression of RB1 indicated a poorer prognosis in our research. Lin et al.^31^ declared ALOX12 related to RB1 in a genome-wide map of humans. Chu et al.^32^ reported that ALOX12 is required for p53-mediated tumor suppression through a distinct ferroptosis pathway. The study challenged several research articles, including ours, and concluded that ALOX12 facilitated the development of tumors^33–35^. Similar to RB1, ALOX12 functions in the ferroptosis pathway through p53. The specific mechanism of RB1, ALOX12, and p53 in ferroptosis needs more research.

A mammalian relative of DNAJ (DNAJB6) belongs to the 40 families of heat shock proteins. Zhang et al.^36^ found an essential axis in OV where DNAJB6 is located in a vital position in the functional axis that is regulated by upstream BRCA1 and that regulated downstream KLF4. As mentioned above, BACH1 could protect BRCA1, which indicated that BACH1 had indirect impacts on DNAJB6. The potential correlation between BACH1 and DNAJB6 revealed that the miR-9/BRCA1/DNAJB6/KLF4/AKT1 axis might have a greater impact on the development of OV than we believed.

STEAP3 was closely related to iron homeostasis. Isobe et al.^9^ reported that STEAP3 maintains tumor growth under hypoferric conditions. This gene joins the regulation of iron homeostasis and inflammatory responses. Men et al.^37^ found that VIMP/ SELENOS was associated with cell death and the cell cycle in insulinoma cells. However, there have been few studies on these genes in OV. What roles they play in OV cell development and apoptosis still needs to be explored.

Although several research studies have declared that ferroptosis plays a crucial role in development and apoptosis, the mechanisms remain elusive. According to the DEGs between the high-risk and low-risk groups, we performed GO enrichment analysis. To our surprise, several immune-related pathways were enriched. The discovery revealed that ferroptosis might exert an influence on OV through tumor immunity. However, few modulations have been reported in the association between ferroptosis and tumor immunity.

Interestingly, the T cells related to immune cells were significantly different in the two subgroups. Moreover, the type II IFN response was the only significantly different immune function in both databases. We speculated that ferroptosis cells promoted the inhibition of the activity of effector T cells by regulating Treg cells. Yin et al.^38^ and Magdalena et al.^39^ declared that increased macrophages and Treg cells in tumor tissue would result in a poorer prognosis in OV patients, consistent with our results. Based on immune gene enrichment, we need to pay more attention to abnormal T cell behaviors in OV patients, and immune treatment deserves more research in future work.

## Limitations

The results were obtained from the data expression matrix. They were not proved by in vivo and clinical studies. Moreover, there might be several essential genes missed in multiple continuous processes.

## Conclusion

Our research constructed a model based on six ferroptosis-related genes. The signature performed well in predicting the prognosis of OV patients both in the training set and test set. The risk score could be an independent factor associated with OS. The potential mechanisms between ferroptosis and tumor immunity in OV are still unclear, and T cell-related immunity changes in OV deserve more investigation.

## Supplementary Information

Supplementary Information.
