# Supplementary material for: A novel ferroptosis related gene signature is associated with prognosis in patients with ovarian serous cystadenocarcinoma
Source: Sci Rep. 2021 Jun 1;11:11486. doi: 10.1038/s41598-021-90126-5 (PMC8169824; doi:10.1038/s41598-021-90126-5)
Supplement: Supplementary file 1 — Supplementary Information. [file 41598_2021_90126_MOESM1_ESM.doc]

A Novel Ferroptosis Related Gene Signature Is Associated With Prognosis In Patients With Ovarian Serous Cystadenocarcinoma

Zhixiang Yu^#3^, Haiyan He^#1^, Yanan Chen^4^, Qiuhe Ji*^2^ and Min Sun*^1^

1 Department of obstetrics and gynecology,Tangdu Hospital, The Air Force Military Medical University, Xi’an Shaanxi, China

2 Department of Endocrinology and Metabolism, Xijing Hospital, The Air Force Military Medical University, Xi’an Shaanxi, China

3 Basic Medicine College, The Air Force Military Medical University, Xi’an Shaanxi, China

4 Department of Medical Oncology, Jinling Hospital, School of Medicine, Nanjing University, Nanjing, Jiangsu, China

^#^Zhixiang Yu and Haiyan He contributed the same to the research

* Correspondence:

Min Sun*

sunmin_dr@163.com

Qiuhe Ji*

qiuheji@hotmail.com


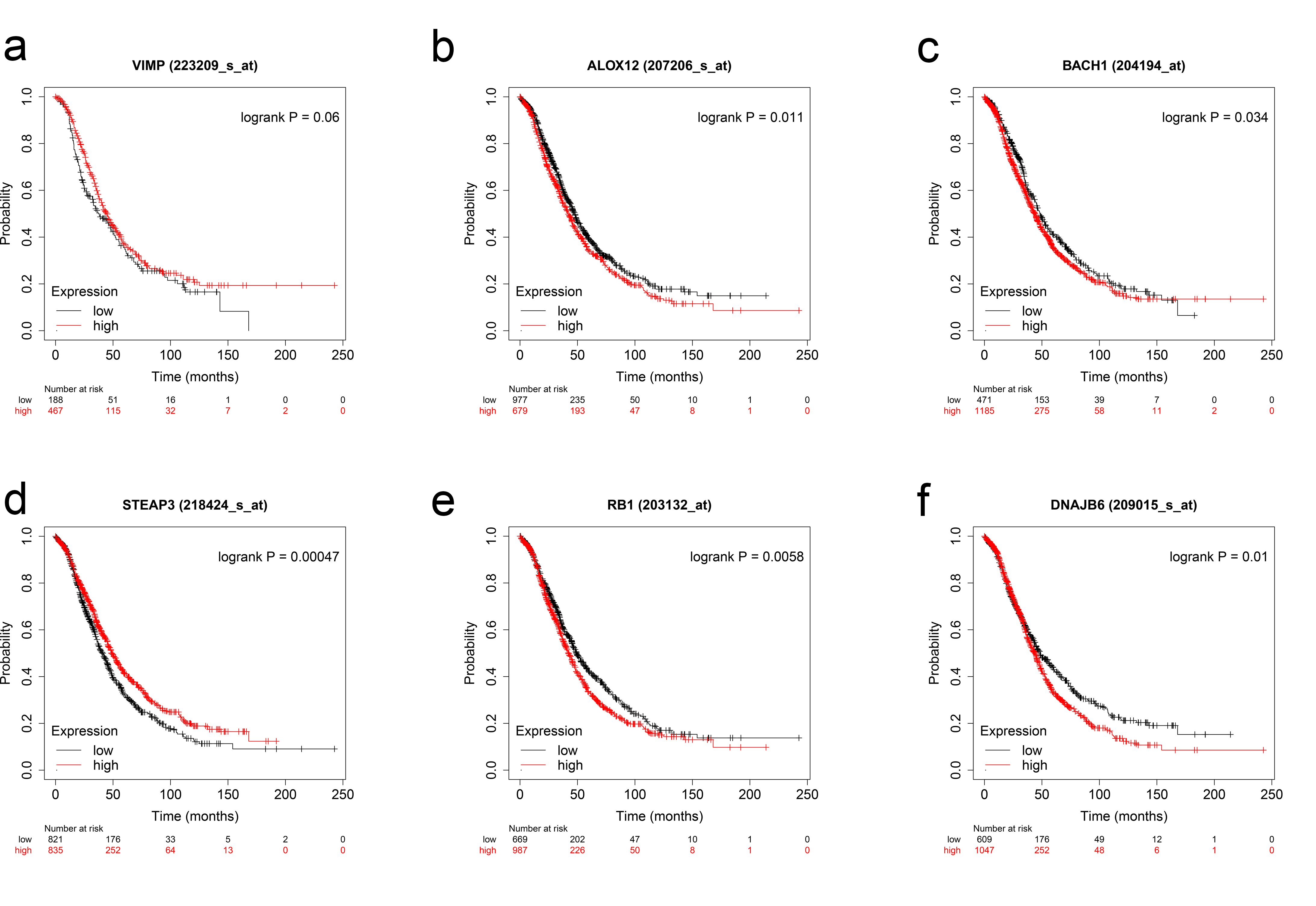


**Supplementary figure 1**. Kaplan-Meier curves for the OS of patients in the high-risk and low-risk groups in the KM plotter ( <https://kmplot.com/analysis/index.php?p=service&cancer=ovar>) in whole dataset. KM plot for(a) VIMP(b) ALOX12(c) BACH1(d) STEAP3(e) RB1 and (f) DNAJB6.


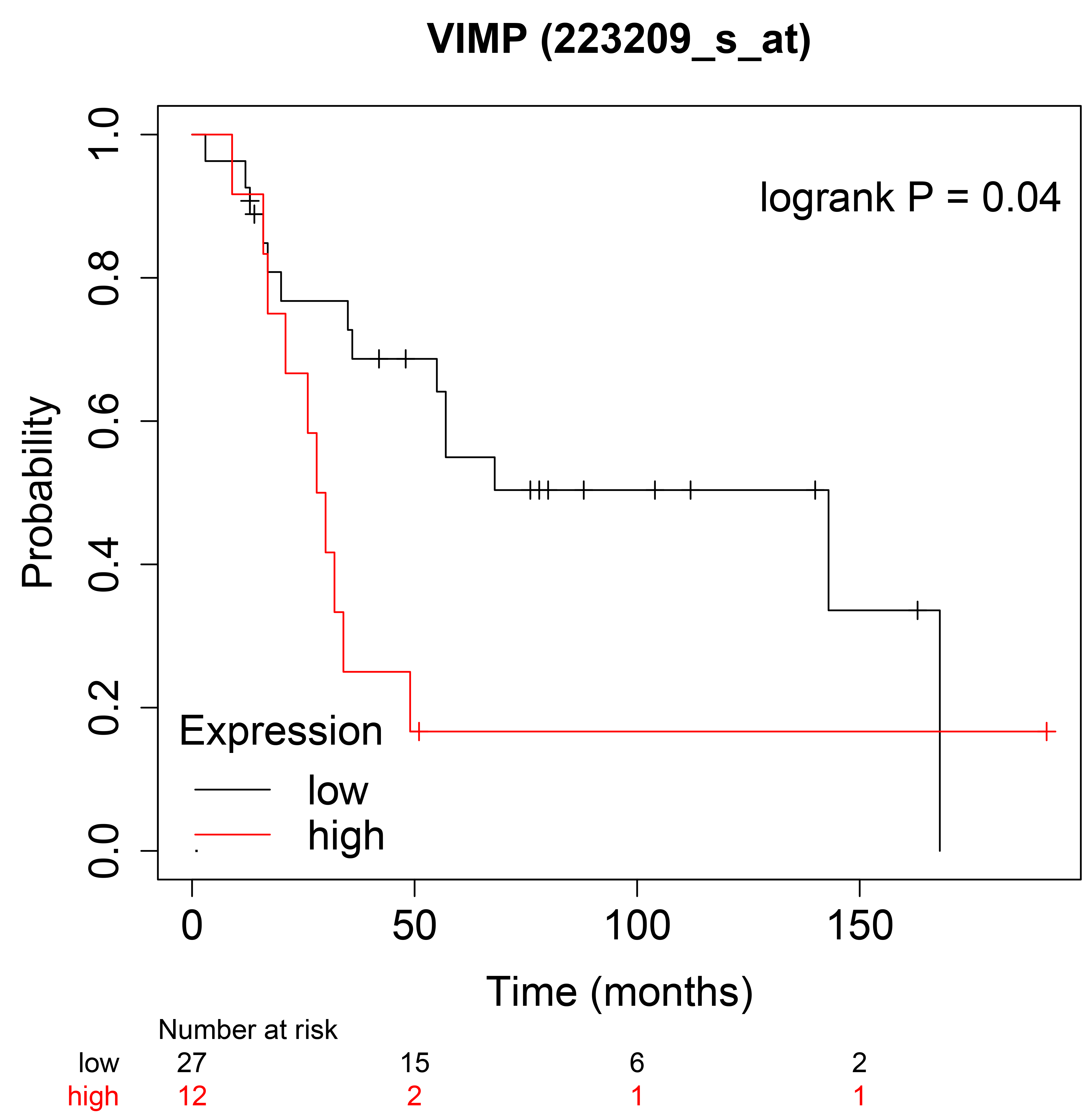


**Supplementary figure 2**. Kaplan-Meier curves for the OS of patients in the high-risk and low-risk groups in the KM plotter ( <https://kmplot.com/analysis/index.php?p=service&cancer=ovar>) in GSE27651 dataset.


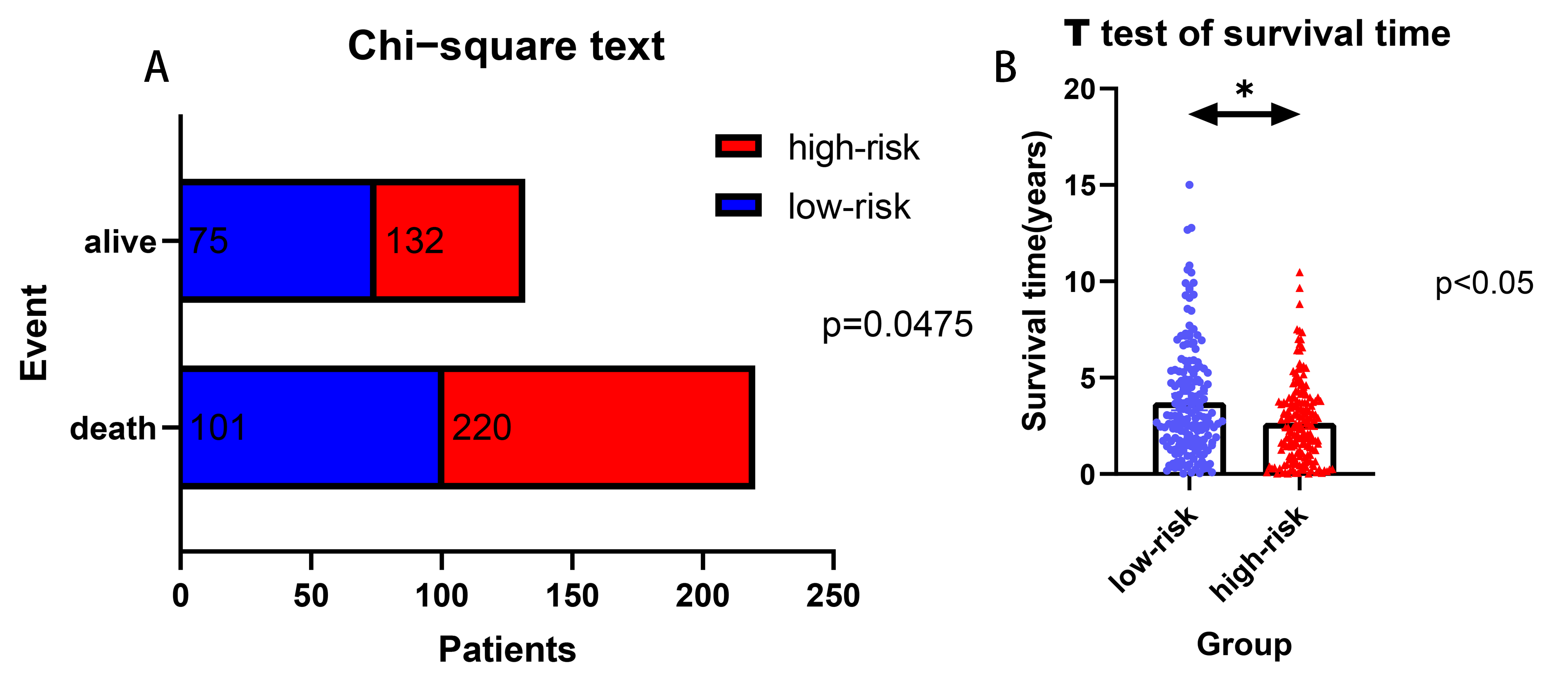


**Supplementary figure 3**. (A) the Chi-square text for the distribution of survival status of the low-risk and high-risk groups. (B) the T text for the difference between the survival time in the low-risk and high-risk groups.


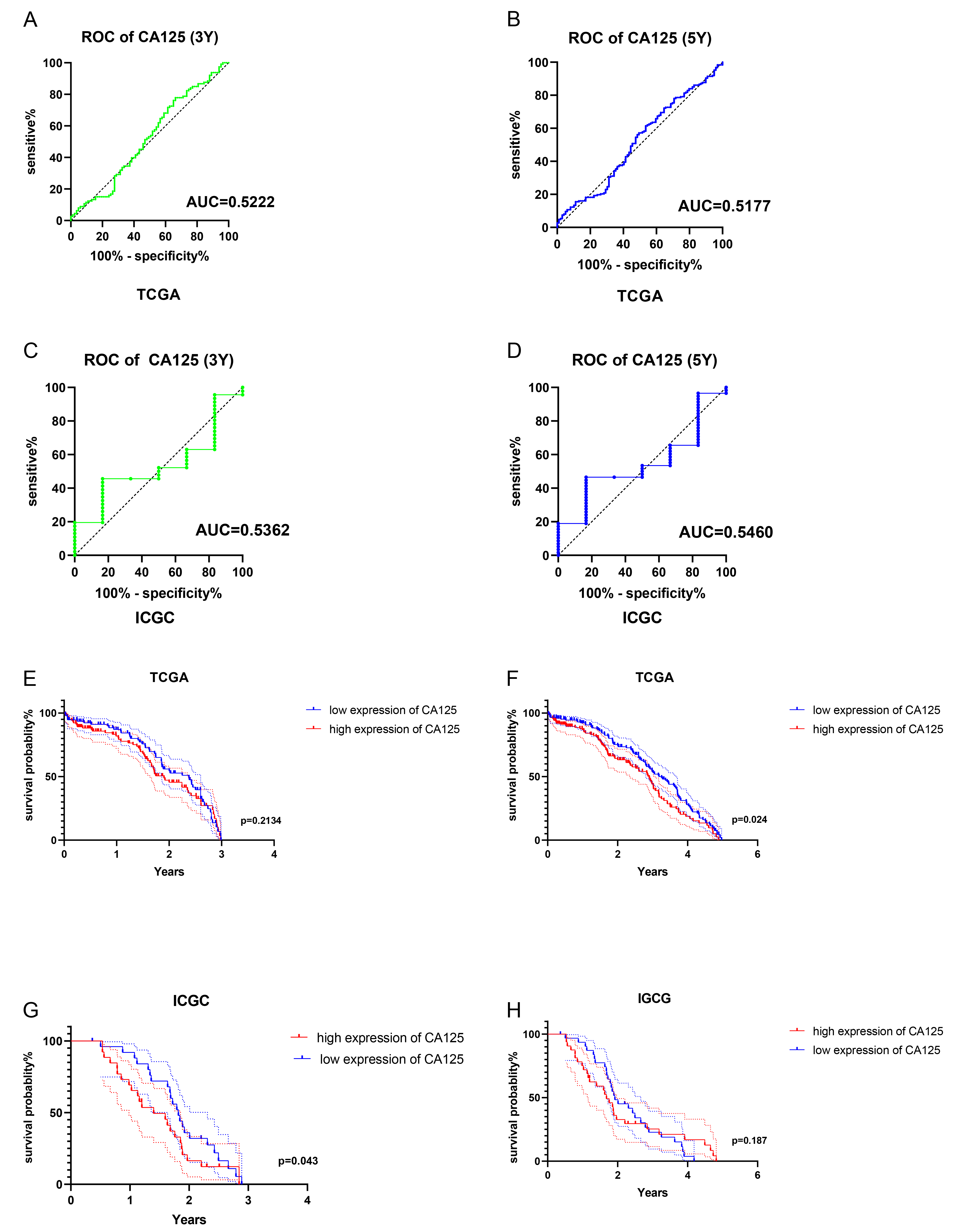


**Supplementary figure 4**. (A) AUC of time-dependent ROC curves verified the MUC16 (CA125) prognostic performance in the TCGA cohort within 3 years. (B) AUC of time-dependent ROC curves verified the MUC16(CA125) prognostic performance in the TCGA cohort within 5 years. (C) AUC of time-dependent ROC curves verified the MUC16 (CA125) prognostic performance in the ICGC cohort within 3 years. (D) AUC of time-dependent ROC curves verified the MUC16 (CA125) prognostic performance in the ICGC cohort within 5 years. (E) Kaplan-Meier curves for the OS of patients in the high MUC16 (CA125) and low MUC16 (CA125) groups in the TCGA cohort within 3 years. (F) Kaplan-Meier curves for the OS of patients in the high MUC16 (CA125) and low MUC16 (CA125) groups in the TCGA cohort within 5 years. (G) Kaplan-Meier curves for the OS of patients in the high MUC16 (CA125) and low MUC16 (CA125) groups in the ICGC cohort within 3 years. (H) Kaplan-Meier curves for the OS of patients in the high MUC16 (CA125) and low MUC16 (CA125) groups in the ICGC cohort within 5 years.


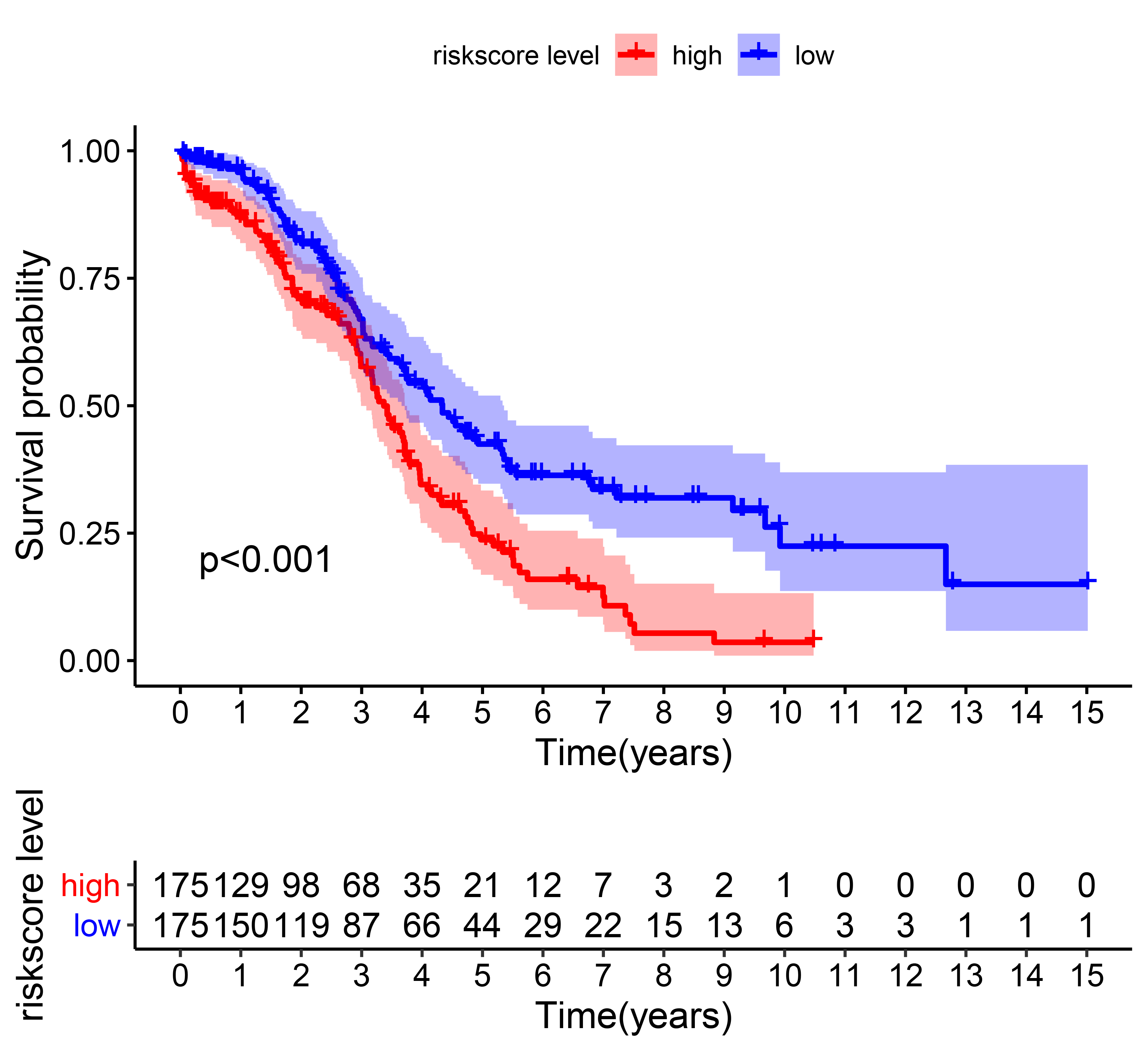


**Supplementary figure 5**. Kaplan-Meier curves for the PFS of patients in the high-risk group and low-risk group in the TCGA cohort

**Supplementary materials. The** Chi-square text for the distribution of TNM stage for high-risk and low-risk groups.

| **Symmetric Measures** | | | | | |
| --- | --- | --- | --- | --- | --- |
|  | | Value | Asymp. Std. Error^a^ | Approx. T^b^ | Approx. Sig. |
| Measure of Agreement | Kappa | -.001 | .001 | -.999 | .318 |
| N of Valid Cases | | 341 |  |  |  |
| a. Not assuming the null hypothesis.  b. Using the asymptotic standard error assuming the null hypothesis. | | | | | |


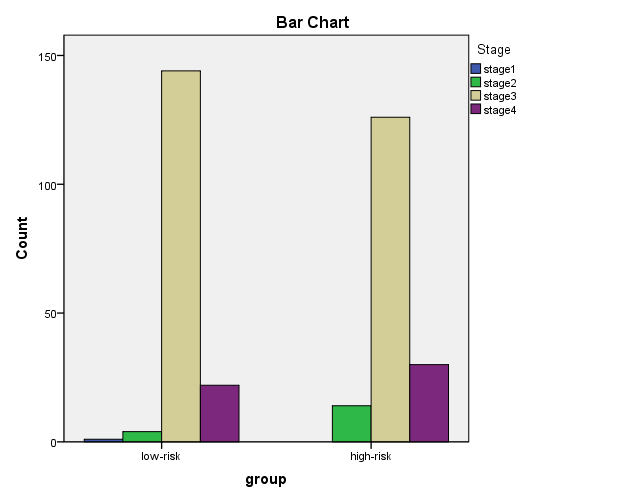


**Supplementary table 1**. part of the KEGG pathway enriched by the DEGs between OV and normal tissues

|  | | Category | CategoryID | GO | Description | LogP | Enrichment | Z-score | | --- | --- | --- | --- | --- | --- | --- | | KEGG Pathway | 24 | hsa04512 | ECM-receptor interaction | -5.4 | 3.6 | 5.8 | | KEGG Pathway | 24 | hsa05332 | Graft-versus-host disease | -5.3 | 4.7 | 6.1 | | KEGG Pathway | 24 | hsa05145 | Toxoplasmosis | -5.2 | 3.1 | 5.5 | | KEGG Pathway | 24 | ko04216 | Ferroptosis | -5.1 | 4.9 | 6.1 | | KEGG Pathway | 24 | hsa05133 | Pertussis | -5.1 | 3.6 | 5.6 | | KEGG Pathway | 24 | hsa04151 | PI3K-Akt signaling pathway | -4.7 | 2 | 4.7 | | KEGG Pathway | 24 | ko05146 | Amoebiasis | -4.7 | 3.2 | 5.2 | | KEGG Pathway | 24 | ko05320 | Autoimmune thyroid disease | -4.6 | 4.1 | 5.4 | | KEGG Pathway | 24 | hsa04216 | Ferroptosis | -4.5 | 4.3 | 5.4 | | KEGG Pathway | 24 | ko05164 | Influenza A | -4.5 | 2.5 | 4.8 | | KEGG Pathway | 24 | ko04151 | PI3K-Akt signaling pathway | -4.4 | 2 | 4.5 | | KEGG Pathway | 24 | ko04015 | Rap1 signaling pathway | -4.4 | 2.3 | 4.6 | | KEGG Pathway | 24 | ko05321 | Inflammatory bowel disease (IBD) | -4.3 | 3.6 | 5.1 | | KEGG Pathway | 24 | hsa04530 | Tight junction | -4.2 | 2.4 | 4.6 | |  |  |  |  |  |  |  |  |  |  |
| --- | --- | --- | --- | --- | --- | --- | --- | --- | --- | --- | --- | --- | --- | --- | --- | --- | --- | --- | --- | --- | --- | --- | --- | --- | --- | --- | --- | --- | --- | --- | --- | --- | --- | --- | --- | --- | --- | --- | --- | --- | --- | --- | --- | --- | --- | --- | --- | --- | --- | --- | --- | --- | --- | --- | --- | --- | --- | --- | --- | --- | --- | --- | --- | --- | --- | --- | --- | --- | --- | --- | --- | --- | --- | --- | --- | --- | --- | --- | --- | --- | --- | --- | --- | --- | --- | --- | --- | --- | --- | --- | --- | --- | --- | --- | --- | --- | --- | --- | --- | --- | --- | --- | --- | --- | --- | --- | --- | --- | --- | --- | --- | --- | --- | --- | --- | --- |
|  |  |  |  |  |  |  |  |  |  |  |  |
|  |  |  |  |  |  |  |  |  |  |  |  |
|  |  |  |  |  |  |  |  |  |  |  |  |
|  |  |  |  |  |  |  |  |  |  |  |  |
|  |  |  |  |  |  |  |  |  |  |  |  |
|  |  |  |  |  |  |  |  |  |  |  |  |
|  |  |  |  |  |  |  |  |  |  |  |  |
|  |  |  |  |  |  |  |  |  |  |  |  |
|  |  |  |  |  |  |  |  |  |  |  |  |
|  |  |  |  |  |  |  |  |  |  |  |  |
|  |  |  |  |  |  |  |  |  |  |  |  |
|  |  |  |  |  |  |  |  |  |  |  |  |
|  |  |  |  |  |  |  |  |  |  |  |  |
|  |  |  |  |  |  |  |  |  |  |  |  |
